# Trends and variations in mantle cell lymphoma incidence from 1995 to 2013: A comparative study between Texas and National SEER areas

**DOI:** 10.18632/oncotarget.22367

**Published:** 2017-11-03

**Authors:** Shuangshuang Fu, Michael Wang, David R Lairson, Ruosha Li, Bo Zhao, Xianglin L Du

**Affiliations:** ^1^ Department of Epidemiology, Human Genetics, and Environmental Science, School of Public Health, The University of Texas Health Science Center in Houston, Houston, Texas 77030, USA; ^2^ Department of Lymphoma and Myeloma, The University of Texas MD Anderson Cancer Center, Houston, Texas 77030, USA; ^3^ Department of Management Policy and Community Health, School of Public Health, The University of Texas Health Science Center in Houston, Houston, Texas 77030, USA; ^4^ Department of Biostatistics, School of Public Health, The University of Texas Health Science Center in Houston, Houston, Texas 77030, USA; ^5^ Department of Medicine, Baylor College of Medicine, Houston, Texas 77030, USA

**Keywords:** mantle cell lymphoma, incidence, SEER, Texas, disparity

## Abstract

**Background:**

Few studies have assessed mantle cell lymphoma (MCL) incidence trends in the U.S. National Cancer Institute's Surveillance, Epidemiology and End Results (SEER) areas. Previous studies were 5 to 9 years old and MCL incidence in Texas remains unknown. This study updated the temporal trends and variations of MCL incidence in the SEER areas and compared them with counterpart data in Texas.

**Results:**

From 1995 to 2013, there were 2, 435 and 5, 193 newly diagnosed MCL patients in Texas and SEER areas. Age-adjusted MCL incidence was 0.91 per 100,000 persons per year in Texas and 1.01 in SEER areas. MCL incidence increased steadily with an annual percent change (APC) of 2.56% in SEER areas and an APC of 2.16% in Texas. In SEER areas, APCs for MCL incidence were significantly different from zero in patients with advanced stage tumor (3.33%), male (2.71%), elderly patients ≥ 80 years old (4.21%) and non-Hispanic white patients (2.83%) (all *P* < 0.05). Similar patterns were found in Texas for both incidence rates and APCs.

**Materials and methods:**

We identified all adult patients with newly diagnosed MCL in Texas Cancer Registry and SEER databases from 1995 to 2013. Age-adjusted incidence rates were calculated and negative binomial regression model was used to assess the factors associated with MCL incidence.

**Conclusions:**

MCL incidence rates increased over time in both Texas and SEER areas, with increases being greater in male, non-Hispanic white, and elderly patient ≥70 years with advanced stage tumors. Texas has similar MCL incidence trends and disparities as the national SEER areas.

## INTRODUCTION

Non-Hodgkin lymphoma (NHL) is the seventh most common cancer and eighth leading cause of cancer death in the U.S. [1]. NHL is also a heterogeneous group of lymphomas; each subtype of NHL has different prognosis and treatment options [2]. Mantle cell lymphoma (MCL) is a rare, aggressive subtype of B-cell NHL, affecting 3% to 6% of patients with NHL [3, 4]. MCL has a poor prognosis, with a median overall survival of 4 to 5 years [5]. Previous studies conducted in the U.S. National Cancer Institute's Surveillance, Epidemiology and End Results (SEER) areas showed that MCL incidence has increased steadily from 1992 [3, 6] when it was established as a distinct type of lymphoma [7].

Previous studies [3, 6] also showed that both prevalence and incidence of MCL were higher in older, male and white populations in the U.S. SEER areas. Only a few studies have been conducted to determine the incidence trends and variations in MCL incidence in the U.S. SEER areas [3, 6, 8], but relatively little is known about its incidence by sociodemographic characteristics and tumor stage. Texas is the second most populated state in the U.S., but is not included in the U.S. SEER registries. In Texas, MCL incidence and disease characteristics remain unexplored and the data were not released until 1995. In SEER areas, the previous MCL incidence studies extended only to 2009, but new data is available, so a new analysis is needed.

Therefore, the purpose of the present study is two-fold. First, we updated the temporal trends and variations of MCL incidence in the U.S. SEER areas and compared them with counterpart data in Texas from 1995 to 2013. Second, we evaluated the variations in MCL incidence by age, gender, race/ethnicity and tumor stage. The present study provides a more comprehensive overview of the MCL incidence and factors associated with the development of MCL at the state and national level, which is important information for unfolding the etiology of MCL and identifying high risk population for disease prevention.

## RESULTS

### Patient characteristics

From 1995 to 2013, there were 2,435 and 5,193 newly diagnosed MCL cases in Texas and SEER areas, accounting for 3.48% and 3.65% of patients with NHL, respectively. The median age at diagnosis was 68 years in Texas and 69 years in SEER areas. Table 1 presents the total number of MCL patients in Texas and SEER, and demographic characteristics for those patients. In Texas, 71.17% of the MCL patients were male, 78.85% were non-Hispanic white, and 61.15% had advanced stage tumor. In SEER areas, 68.75% were male, 80.53% were non-Hispanic white, and 75.47% had advanced stage tumor. The percentage of MCL patients with advanced stage tumor in SEER areas was 14.32% higher than that in Texas.

**Table 1:** Demographic characteristics of MCL patients in Texas and SEER areas, 1995–2013

	Number and column % of cancer cases			
	Texas		SEER	
**Year of diagnosis**				
1995–1999	405	16.63	949	18.27
2000–2004	463	19.01	960	18.49
2005–2008	726	29.82	1,510	29.08
2009–2013	841	34.54	1,774	34.16
**Age category**				
< 50	200	8.21	368	7.09
50–59	428	17.58	932	17.95
60–69	736	30.23	1,399	26.94
70–79	704	28.91	1,498	28.85
≥ 80	367	15.07	996	19.18
**Gender**				
Male	1,733	71.17	3, 570	68.75
Female	702	28.83	1,623	31.25
**Race**				
Non-Hispanic Black	108	4.44	220	4.24
Non-Hispanic White	1,920	78.85	4,182	80.53
Hispanic	366	15.03	445	8.57
Other	41	1.68	346	6.66
**Marital Status**				
Single	141	5.79	526	10.13
Married (including common law)	933	38.32	3,257	62.72
Divorced, separated, or widowed	290	11.91	1,076	20.72
Unknown	1,071	43.98	334	6.43
**Tumor Stage**				
Localized	302	12.40	546	10.51
Regional	181	7.43	428	8.24
Advanced	1,489	61.15	3,919	75.47
Unknown	463	19.01	300	5.78
**Total**	2,435		5,193	

### MCL incidence rate and relative risk (RR) Over 19 years in Texas and SEER areas

Table 2 presents MCL incidence rates and RRs in Texas and SEER. From 1995 to 2013, overall age-adjusted MCL incidence was 0.91 per 100,000 persons per year in Texas and 1.01 per 100,000 persons per year in SEER areas. In Texas, the risk of MCL increased with age and was highest in patients aged ≥ 70 years when compared to those aged under 50 (RR for 70–79 years: 32.27, 95% CI: 27.30–38.15; RR for 80+ years: 32.04, 95% CI: 26.69–38.47). The risk of MCL was higher in male (RR: 3.00, 95% CI: 2.72–3.30) compared to females. Both non-Hispanic white (RR: 2.47, 95% CI: 2.03–3.01) and Hispanic (RR: 1.42, 95% CI: 1.15–1.77) had a higher risk of developing MCL compared to non-Hispanic Black. The incidence rate for patients with advanced stage tumor was about five times that of localized stage (RR: 4.91, 95% CI: 4.30–5.59). In SEER areas, the risk of MCL increased with age and was highest in patients aged ≥ 80 years (RR: 42.01, 95% CI: 35.75–49.36). The risk of MCL was also higher in male (RR: 2.65, 95% CI: 2.43–2.89), non-Hispanic white (RR: 2.24, 95% CI: 1.92–2.61), and patients with advanced stage tumor (RR: 6.88, 95% CI: 6.11–7.73).

**Table 2:** Age-adjusted incidence rates and rate ratios of MCL in Texas and SEER areas, 1995–2013

	Texas		SEER	
	Incidence^a^ (95% CI)	RR^b^ (95% CI)	Incidence^a^ (95% CI)	RR^b^ (95% CI)
**Year of diagnosis**				
1995–1999	0.70 (0.63–0.77)	1.00 (Reference)	0.80 (0.75–0.85)	1.00 (Reference)
2000–2004	0.91 (0.82–0.99)	1.26 (1.08–1.46)	0.94 (0.88–1.00)	1.14 (1.00–1.30)
2005–2008	1.01 (0.93–1.08)	1.39 (1.21–1.60)	1.09 (1.03–1.15)	1.26 (1.11–1.43)
2009–2013	1.00 (0.93–1.07)	1.39 (1.21–1.60)	1.15 (1.09–1.20)	1.34 (1.19–1.52)
**PC 1995–2013, %**	53.57		88.42	
**APC 1995–2013, %**	2.16^*^		2.56^*^	
**Age (years)**				
< 50	0.11 (0.10–0.13)	1.00 (Reference)	0.11 (0.10–0.13)	1.00 (Reference)
50–59	0.89 (0.81–0.98)	7.88 (6.60–9.40)	1.03 (0.97–1.10)	8.49 (7.25–9.95)
60–69	2.42 (2.25–2.60)	20.29 (17.17–23.97)	2.48 (2.35–2.61)	21.23 (18.23–24.73)
70–79	3.65 (3.38–3.93)	32.27 (27.30–38.15)	3.99 (3.79–4.20)	36.46 (31.31–42.46)
≥ 80	3.38 (3.04–3.74)	32.04 (26.69–38.47)	4.15 (3.90–4.42)	42.01 (35.75–49.36)
**Gender**				
Female	0.48 (0.45–0.52)	1.00 (Reference)	0.57 (0.54–0.59)	1.00 (Reference)
Male	1.46 (1.39–1.53)	3.00 (2.72–3.30)	1.58 (1.52–1.63)	2.65 (2.43–2.89)
**Race**				
Non-Hispanic Black	0.42 (0.34–0.51)	1.00 (Reference)	0.49 (0.43–0.57)	1.00 (Reference)
Non-Hispanic White	1.08 (1.03–1.13)	2.47 (2.03–3.01)	1.18 (1.15–1.22)	2.24 (1.92–2.61)
Hispanic	0.65 (0.58–0.72)	1.42 (1.15–1.77)	0.79 (0.72–0.87)	1.42 (1.18–1.69)
Other	0.51 (0.35–0.71)	1.17 (0.81–1.68)	0.56 (0.50–0.62)	1.07 (0.89–1.28)
**Tumor stage**				
Localized	0.11 (0.10–0.13)	1.00 (Reference)	0.11 (0.10–0.12)	1.00 (Reference)
Regional	0.07 (0.06–0.08)	0.60 (0.50–0.72)	0.08 (0.08–0.09)	0.76 (0.66–0.89)
Advanced	0.56 (0.53–0.59)	4.91 (4.30–5.59)	0.76 (0.73–0.78)	6.88 (6.11–7.73)
Unknown	0.17 (0.16–0.19)	1.53 (1.31–1.78)	0.06 (0.05–0.07)	0.55 (0.47–0.65)

95% CI indicates 95% confidence interval.

^a^Incidence rate was per 100,000 persons per year and were age-adjusted to the U.S. 2000 Census using the SEER^*^Stat statistical program.

^b^Relative risk was adjusted for year of diagnosis, age, gender, and tumor stage.

^*^Significantly different from 0, *P* < 0.05.

Abbreviations. PC, percent change. APC, annual percent change.

### MCL incidence trend over 19 years by demographic factors

**Figure 1:**
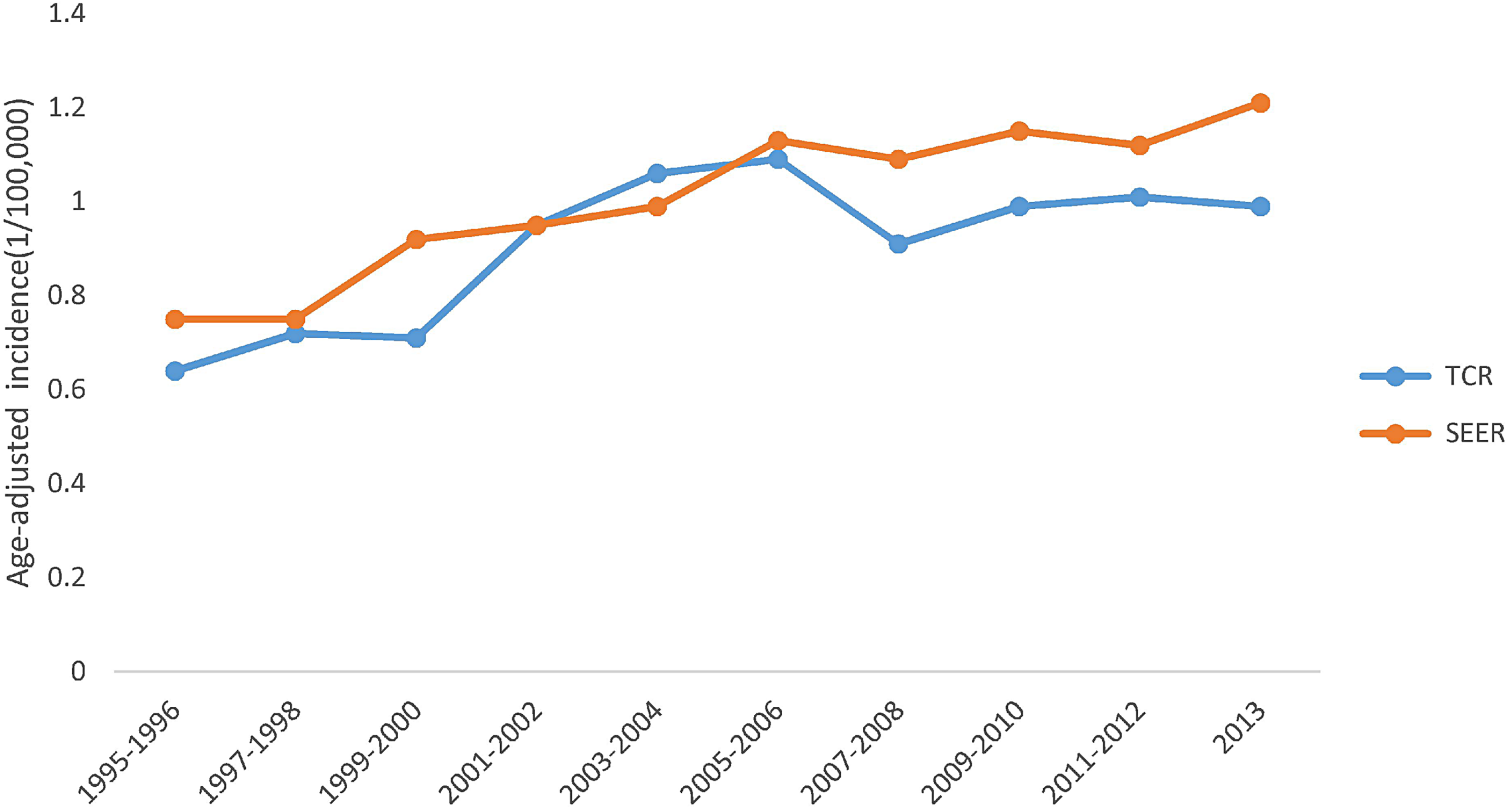
MCL age-adjusted incidence rates over time, 1995–2013

**Figure 2:**
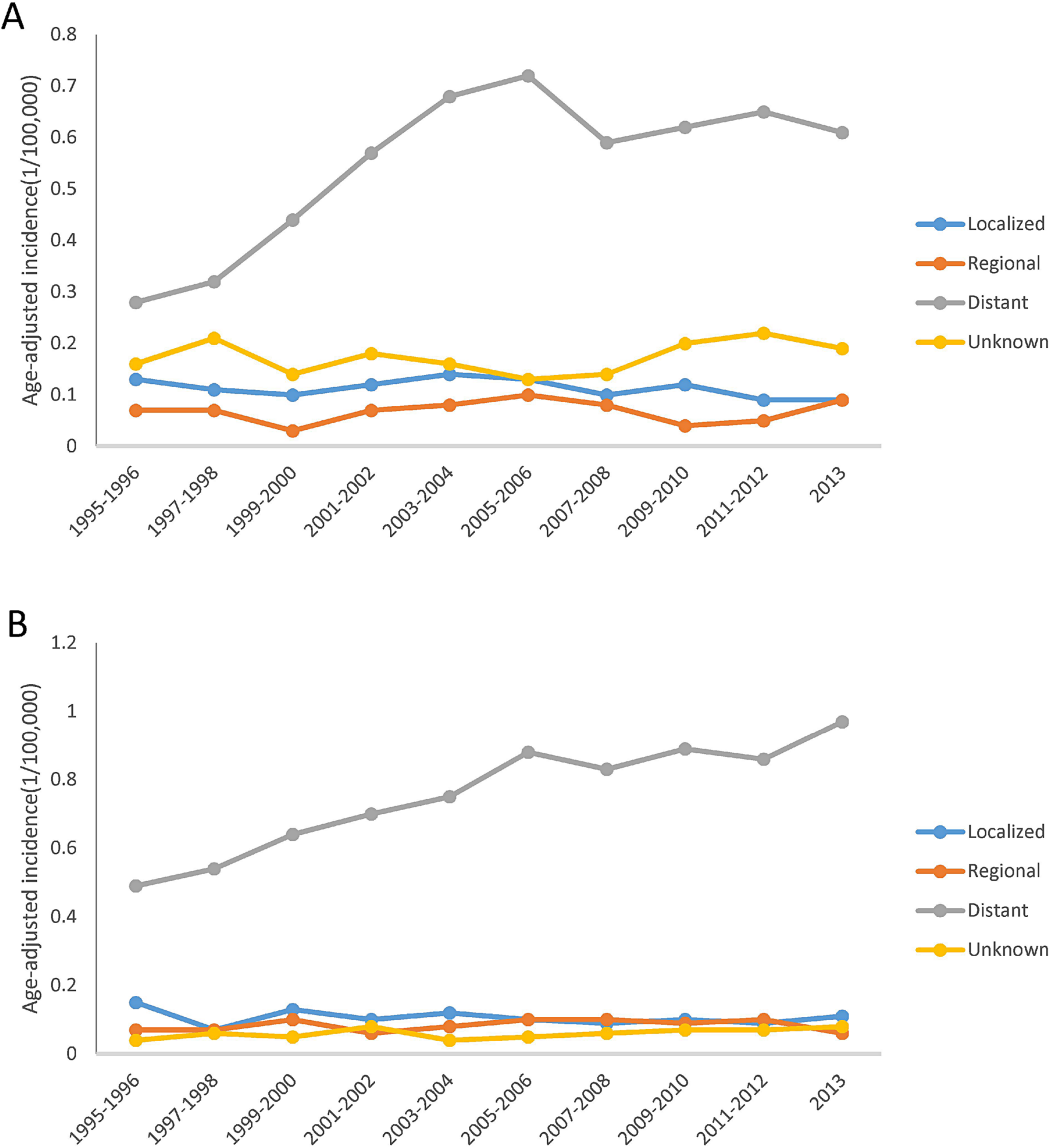
MCL age-adjusted incidence rates over time by tumor stage, 1995–2013 (**A**) Texas. (**B**) SEER.

**Figure 3:**
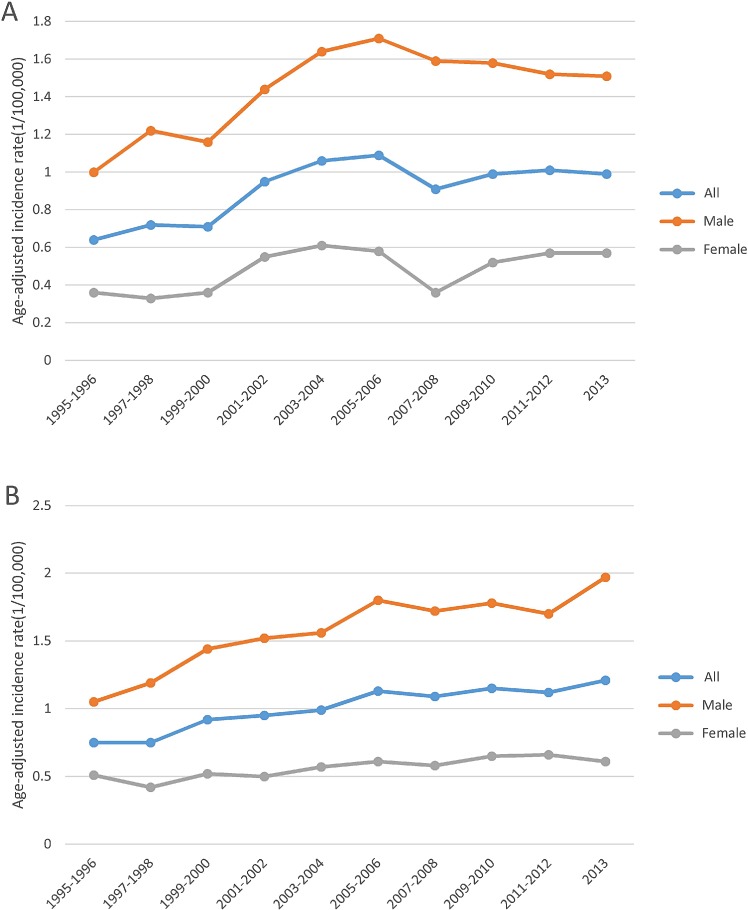
MCL age-adjusted incidence trends by sex, 1995–2013 (**A**) Texas. (**B**) SEER.

**Figure 4:**
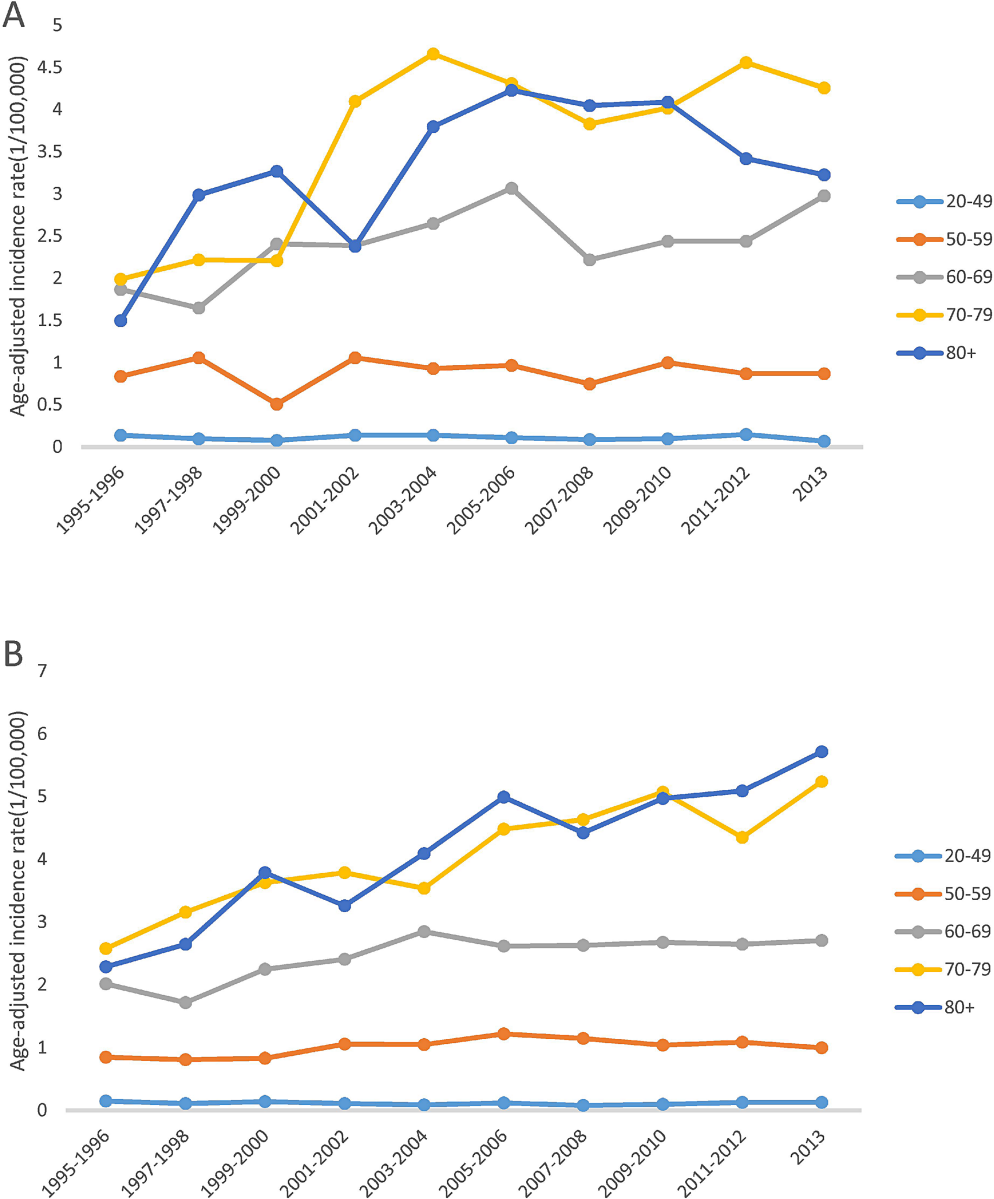
MCL age-adjusted incidence trends by age group, 1995–2013 (**A**) Texas. (**B**) SEER.

**Figure 5:**
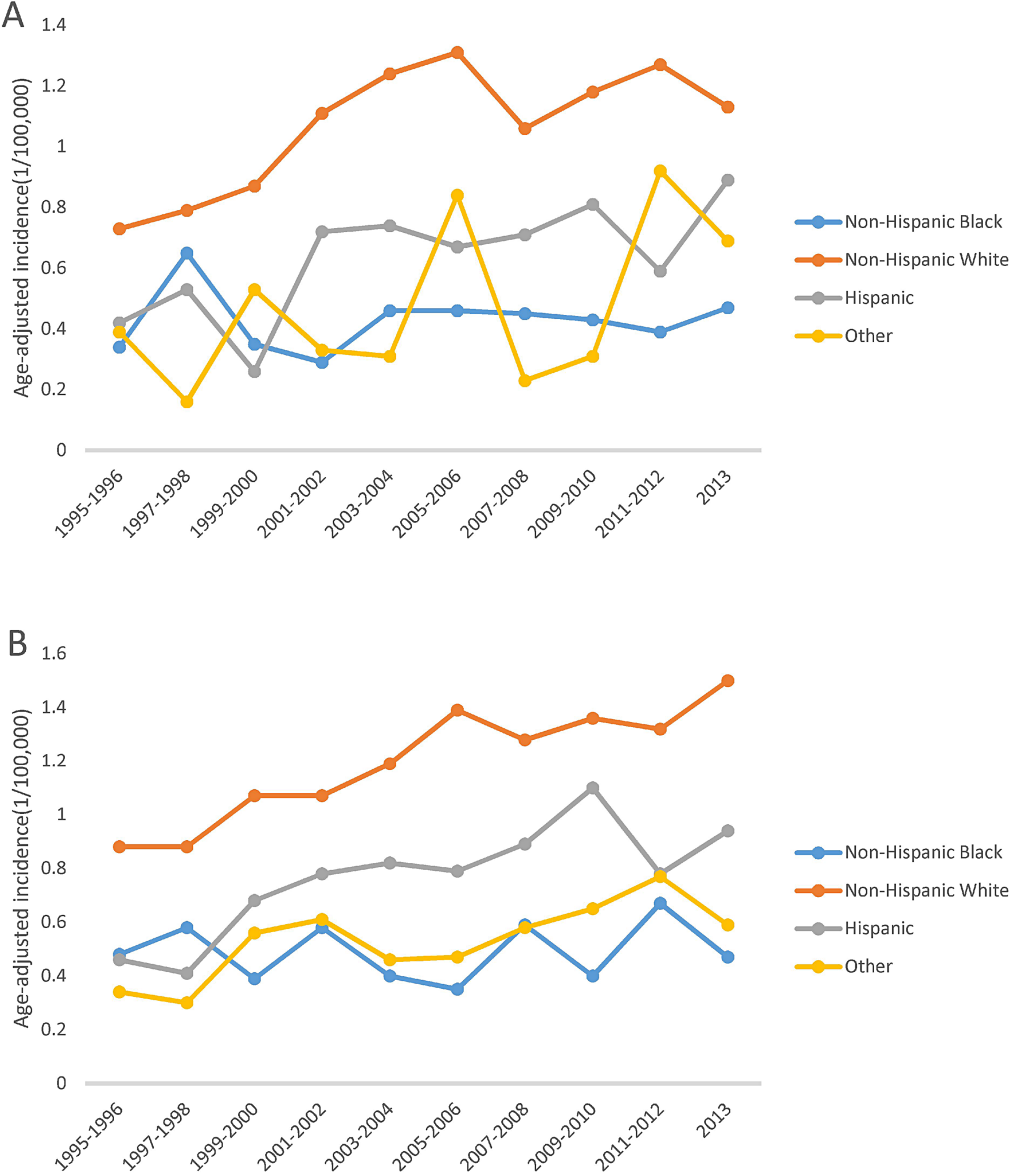
MCL age-adjusted incidence trends by race/ethnicity, 1995–2013 (**A**) Texas. (**B**) SEER.

Figure 1 shows that overall MCL incidence increased steadily in SEER areas with an annual percent change (APC) of 2.56% (*p* < 0.05), but in Texas, the incidence increased from 1995 to 2006, then became stable from 2007 to 2013, with an APC of 2.16% (*p* < 0.05). Figure 2 shows that in both SEER and Texas, MCL incidence rates for patients with advanced stage tumors increased significantly (APC for SEER: 3.33% vs. Texas: 3.40%, all *p* < 0.05). MCL incidence increase was steeper from 1995 to 2005 and then plateaued after 2007. Figure 3 presents MCL incidence trends by gender. In SEER areas, MCL incidence rates experienced a greater increase for male (APC 2.71%, *p* < 0.05) compared to female (APC 1.99%, *p* < 0.05) over the past two decades. However, in Texas, MCL incidence rates in female (APC 2.22%, *p* < 0.05) increased more compared to male (APC 2.02%, *p* < 0.05). Figure 4 presents MCL incidence rates by age group. In SEER areas, three age groups had significant increases in MCL incidence; patients aged 60–69 years had an APC of 1.74% (*p* < 0.05), patients aged 70–79 had an APC of 3.29% (*p* < 0.05), and patients aged ≥ 80 years had an APC of 4.21% (*p* < 0.05). In Texas, only patients aged 70–79 years had a significant increase in MCL incidence with an APC of 3.62% (*p* < 0.05). Figure 5 presents MCL incidence rates by race/ethnicity. MCL incidence rates increased significantly in non-Hispanic white and Hispanic population for both SEER areas (non-Hispanic white APC: 2.83%, Hispanic APC: 3.50%, all *p* < 0.05) and Texas (non-Hispanic white APC: 2.53%, Hispanic: 2.50%, all *p* < 0.05).

### Incidence and trend analyses broken down by year of diagnosis and age of diagnosis for non-hispanic white, male and patients with advanced stage tumor

Table 3 presents the subgroup incidence and trend analyses for non-Hispanic white, male and patients with advanced stage tumor. These three groups of patients had higher incidence rates compared to their reference groups and had experienced higher increase in incidence rates. Therefore, subgroup analyses by year of diagnosis and age at diagnosis were conducted for those three groups and compared between SEER areas and Texas. MCL incidence rate increases in non-Hispanic white and male population were higher in SEER areas compared to Texas (non-Hispanic white APC, SEER: 2.83% vs. Texas: 2.53%; male APC, SEER: 2.71% vs. Texas: 2.02%, all *p* < 0.05). However, MCL incidence increase in patients with advanced stage tumor in SEER areas was slightly lower compared to that in Texas (APC in SEER: 3.33% vs. Texas: 3.40, all *p* < 0.05). We also found that in SEER areas, elderly males had the highest MCL incidence rates (IRs) among all subgroups with high MCL incidence (70–79 years, IR: 6.24, 95% CI: 5.86–6.63; 80+ years, IR: 7.22, 95% CI: 6.66–7.81), indicating that substantial disparities in MCL incidence exist in gender and age groups. Texas had similar patterns in incidence rate distribution by demographic characteristics (male 70–79 years, IR: 5.55, 95% CI: 5.06–6.08; male 80+ years, IR: 6.06, 95% CI: 5.31–6.89), but the rates were slightly lower compared to SEER areas.

**Table 3:** MCL incidence rates for non-Hispanic white, male and patients with advanced stage tumor, SEER and Texas areas, 1995–2013

SEER						
	non-Hispanic White		Male		Advanced stage tumor	
	Incidence^a^ 95% CI	Count	Incidence^a^ 95% CI	Count	Incidence^a^ 95% CI	Count
**Year of diagnosis**						
1995–1999	0.88 (0.82–0.94)	849	1.20 (1.11–1.3)	622	0.55 (0.51–0.59)	653
2000–2003	1.05 (0.98–1.12)	856	1.52 (1.4–1.64)	679	0.70 (0.65–0.76)	721
2004–2008	1.25 (1.19–1.32)	1,363	1.71 (1.6–1.81)	1,044	0.83 (0.78–0.88)	1,157
2009–2013	1.31 (1.24–1.37)	1,549	1.78 (1.68–1.89)	1,225	0.89 (0.84–0.94)	1,388
1995–2013	1.13 (1.1–1.17)	4,617	1.58 (1.52–1.63)	3,570	0.76 (0.73–0.78)	3,919
**PC 1995–2013**	99.72		109.82		156.66	
**APC 1995–2013**	2.83^*^		2.71^*^		3.33^*^	
**Age at diagnosis**						
20–49	0.14 (0.12–0.16)	262	0.16 (0.14–0.18)	261	0.09 (0.08–0.1)	289
50–59	1.25 (1.16–1.35)	729	1.53 (1.42–1.65)	675	0.84 (0.78–0.91)	762
60–69	2.87 (2.7–3.05)	1,088	3.76 (3.53–4.01)	1,001	1.94 (1.83–2.06)	1,098
70–79	4.69 (4.43–4.96)	1,244	6.24 (5.86–6.63)	1,015	2.93 (2.76–3.11)	1,100
80+	4.72 (4.41–5.04)	859	7.22 (6.66–7.81)	618	2.80 (2.59–3.02)	670

**Table:** 

Texas						
	non-Hispanic White		Male		Advanced stage tumor	
	Incidence^a^ 95% CI	Count	Incidence^a^ 95% CI	Count	Incidence^a^ 95% CI	Count
**Year of diagnosis**						
1995–1999	0.79 (0.71–0.88)	326	1.12 (1.00–1.27)	289	0.34 (0.29–0.39)	195
2000–2003	1.07 (0.97–1.19)	377	1.39 (1.24–1.55)	318	0.56 (0.50–0.63)	288
2004–2008	1.19 (1.10–1.30)	574	1.66 (1.52–1.81)	535	0.66 (0.60–0.72)	474
2009–2013	1.21 (1.11–1.31)	643	1.54 (1.42–1.68)	591	0.63 (0.58–0.69)	532
1995–2013	1.08 (1.03–1.13)	1,920	1.46 (1.39–1.53)	1,733	0.56 (0.53–0.59)	1,489
**PC 1995–2013**	69.42		66.72		144.74	
**APC 1995–2013**	2.53^*^		2.02^*^		3.40^*^	
**Age at diagnosis**						
20–49	0.14 (0.12–0.17)	136	0.17 (0.15–0.2)	154	0.07 (0.06–0.08)	119
50–59	1.02 (0.91–1.15)	301	1.37 (1.23–1.53)	323	0.54 (0.48–0.61)	262
60–69	2.92 (2.69–3.17)	591	3.84 (3.52–4.17)	552	1.51 (1.38–1.66)	459
70–79	4.29 (3.95–4.66)	583	5.55 (5.06–6.08)	469	2.21 (2.01–2.44)	427
80+	3.80 (3.38–4.24)	309	6.06 (5.31–6.89)	235	2.04 (1.78–2.33)	222

^a^Incidence rate was per 100,000 persons per year and age-adjusted to the U.S. 2000 Census using the SEER^*^Stat statistical program.

^*^Significantly different from 0, *P* < 0.05.

### Overall survival (OS) for MCL patients in SEER and TCR areas

**Figure 6:**
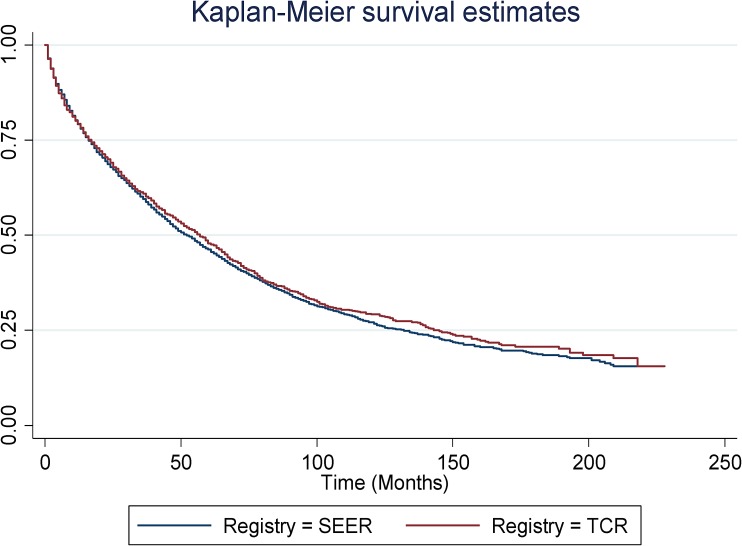
Kaplan-Meier survival estimates for SEER and TCR

**Figure 7:**
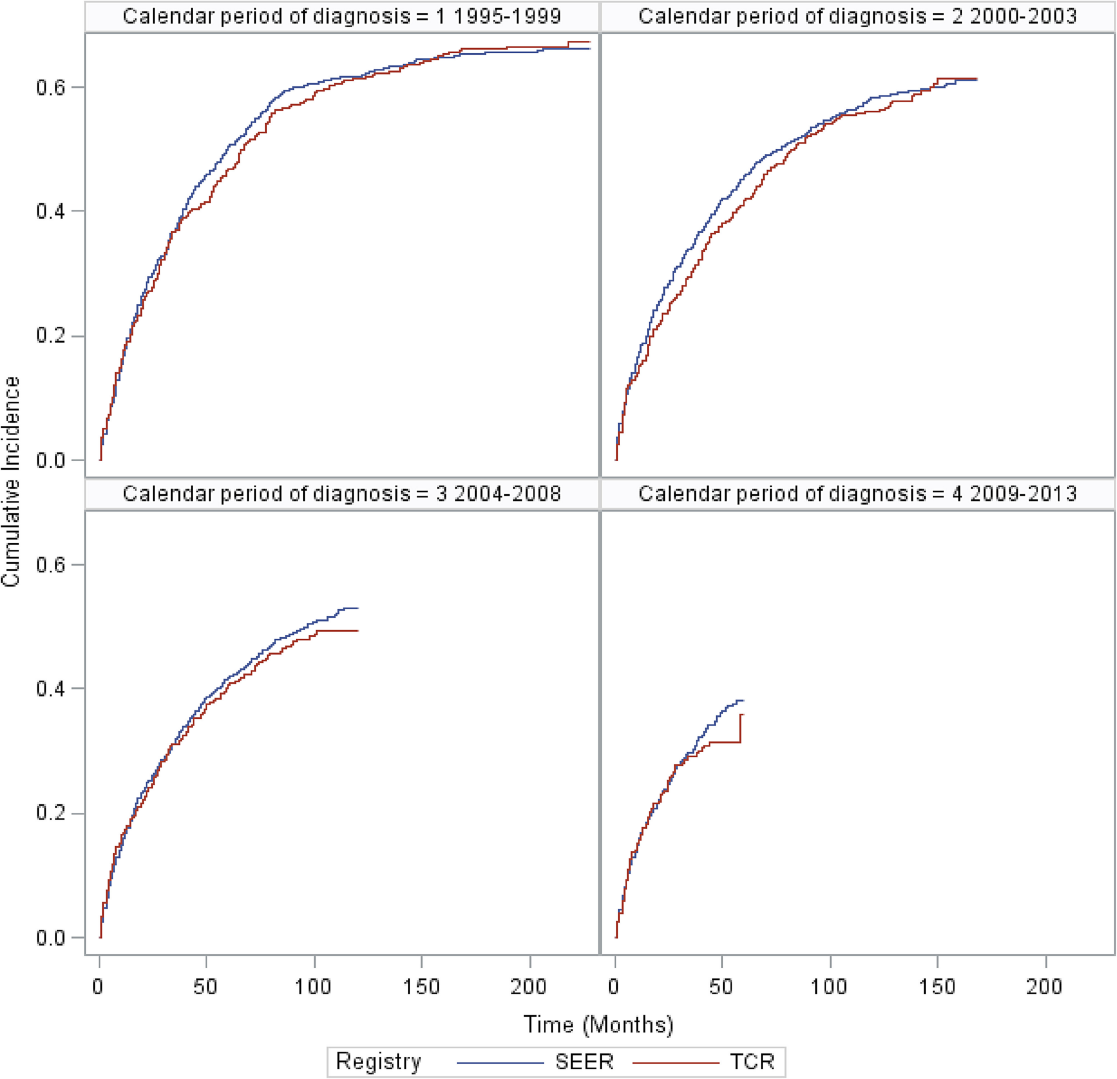
Cumulative incidence of MCL death for SEER and TCR areas over calendar period of diagnosis

**Figure 8:**
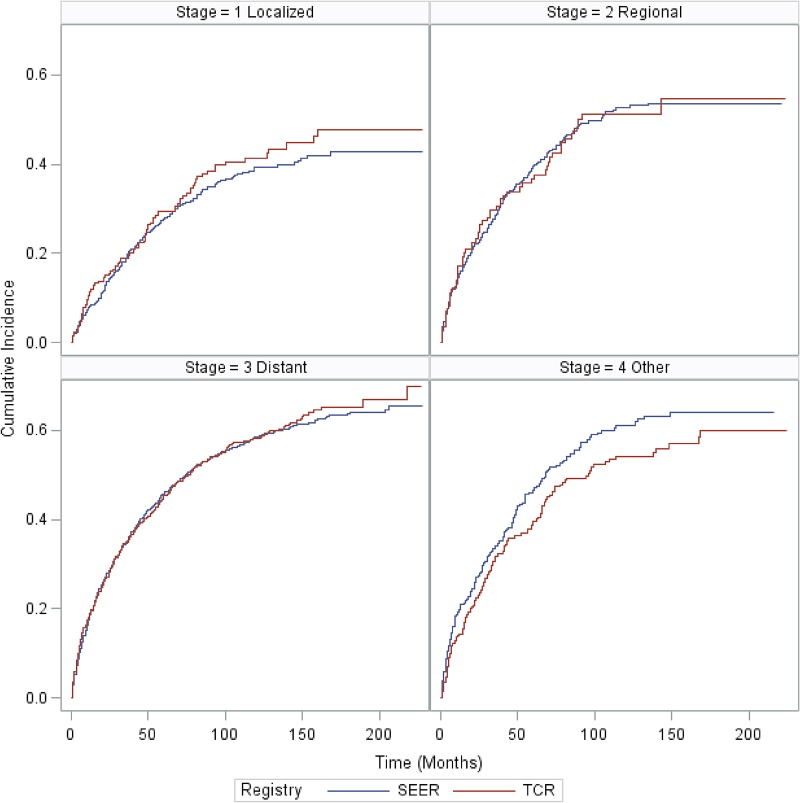
Cumulative incidence of MCL death for SEER and TCR areas by tumor stage

Median OS was 52 months and 57 months for MCL patients in SEER and TCR areas, respectively. There was no statistically significant difference in the OS between SEER and TCR (Figure 6). Figure 7 presents the cumulative incidence of death by calendar period of diagnosis. The cumulative incidence of death decreased over time, but for patients diagnosed in each time period, there were no significant differences between the two registries. Figure 8 presents the cumulative incidence of death by tumor stage. The cumulative incidence of death was lowest for patients with localized stage tumor and highest for patients with distant stage tumor. Nevertheless, for patients diagnosed with each stage tumor, there was no significant difference in the cumulative incidence of death between the two registries.

## DISCUSSION

This study assessed the incidence trends and variations for MCL over the past two decades. It is the first study to compare the results of MCL incidence trends in Texas to those of SEER areas. Patient characteristics by age, gender, race/ethnicity and tumor stage had similar patterns between Texas and SEER areas. MCL incidence disparities by gender and race/ethnicity existed in both Texas and SEER areas. The MCL incidence rates in Texas were slightly lower than that in SEER areas. One of the reasons for a lower rate in Texas is a possible under-ascertainment of Texas cases. Another reason is the increasing Texas population with more young people moving to Texas. However, all incidence rates were age-adjusted to the 2000 U.S. standard population to remove potential confounding by age differences.

This study showed that in SEER areas, MCL incidence rates increased steadily and significantly in elderly patients aged 60 years or older. This finding is consistent with previous findings on higher MCL incidence in the elderly population in the U.S. SEER areas [3]. Texas MCL incidence rates increased significantly only in patients aged 70 to 80 years old. The reasons why MCL increased more in the elderly population remain unknown, but elderly people could have more chronic diseases and were more likely to seek medical services, increasing opportunistic findings for MCL [9, 10]. This information is important for informing future drug development and clinical management for MCL patients, since elderly patients are more likely to have multiple chronic complications and suffer from drug-related side effects and the elderly population tends to be underrepresented in clinical trials [11, 12].

This study also revealed that in SEER areas, the age-adjusted incidence rate for advanced stage MCL was the highest among all tumor stages. The increase of MCL incidence in advanced stage MCL was also stronger than that in any other tumor stages. MCL incidence increased steadily from 1995 to 2006, and then decreased slightly from 2006 to 2008; after 2008, MCL incidence continued to increase steadily. Similar results were observed in Texas, except that Texas had a lower incidence rate in advanced stage tumor and experienced a slight drop in incidence from 2004 to 2013. This increasing trend over the past 20 years can be explained by a few reasons. MCL patients at early stage are usually asymptomatic, therefore, it is difficult to detect MCL at an early stage. In recent years, doctors are more aware of the behavior of MCL and are doing gastrointestinal endoscopy evaluations for stage 1 or 2 patients which were not initially done, some MCL patients were upstaged because of upper/lower gastrointestinal endoscopies even if the patients were asymptomatic. Also, better tools are available to detect MCL, such as positron emission tomography scanning and peripheral blood markers analyses by flow cytometry. The percentage of advanced stage tumor in SEER areas is 14.32% higher than that in Texas. The difference is largely due to a larger percent of patients in Texas with unknown tumor stage (Table 1).

MCL was found in predominantly male and non-Hispanic white populations in Texas and in SEER areas. The reason why MCL occurred more in male and non-Hispanic white is unknown, but studies have shown that men and women are exposed to different occupational hazards [13, 14]. Genetic studies have shown that t(11; 14) translocation is a molecular hallmark in MCL development [15]. The prevalence of translocation t(11; 14) in healthy populations is low, and tends to be slightly higher in males and whites compared to females and blacks [16, 17]. Future studies may address the gender and race/ethnicity disparities in MCL incidence rates by evaluating whether there are disparities in genetic mutation and environmental exposures to toxic agents or radiation by gender and race/ethnicity because both genetic and environmental factors are highly relevant to the development of tumors.

Our study showed that MCL incidence in the elderly and white population with advanced stage tumor has been increasing. Given the fact that age is an important factor affecting the treatment regimen selection, future studies on therapeutic agents should target more in the elderly patient group. The study showed the decreased mortality rates over time in both SEER and TCR areas, indicating that patients have benefited from the development of novel agents. More detailed analyses on survival trends and the impact of novel agents on the sur*vivo*rship are warranted.

A series of novel agents have been approved by the FDA to treat MCL since the late 1990s, including rituximab, bortezomib, temsirolimus, bendamustine, lenalidomide, and ibrutinib [18–22]. The treatment regimens for MCL patients differ by age. For symptomatic elderly patients, non-intensive regimens are recommended as first-line treatment regimens, the most common two regimens are rituximab plus cyclophosphamide, doxorubicin, vincristine, and prednisone regimen (R-CHOP) and rituximab plus bendamustine regimen [5, 19]. For young patients under 65, aggressive therapy regimens have revealed promising outcomes from several studies and are recommended as first-line treatment, including two major aggressive regimens (Hyper-CVAD regimen and Nordic regimen) [23, 24]. These intensive treatment regimens are mostly recommended for healthier and younger patients because the healthier and younger patients have better tolerance. Therefore, our study findings indicate that in the future, drug development for MCL should focus more on the elderly population.

The study has two important limitations. First, since these two databases only cover around 22% of the US population, the results may not be generalizable to the entire U.S. population. Second, a number of important known risk factors such as genetics and family history are not available in the data and thus cannot be studied. The differences in these factors may contribute to the observed differences in MCL incidence. The study has important strengths. First, SEER and TCR data include highly valid patient information, tumor variables and sur*vivo*rship. Second, both TCR and SEER cancer registries have been running for many years, which allow us to generate time trend analysis in the MCL incidences. Adding information from Texas provides a more comprehensive understanding of MCL disease characteristics by different geographic locations.

## MATERIALS AND METHODS

### Data sources

All newly diagnosed MCL cases were obtained through Texas Cancer Registry (TCR) and SEER public-use data from 1995 to 2013. TCR is a statewide registry measuring Texas cancer burden, diagnosis, treatment and sur*vivo*rship; it is also one of the largest cancer registries in the United States covering around 8% of U.S. population [25]. From 1992 to 1999, SEER had 13 registries and covered 14% of US population [26]. After 2000, SEER registries were expanded to 18 registries (original 13 registries and extra 5 registries included) capturing around 26% of the U.S. population [26]. Because our study compared the MCL incidence between Texas and SEER from 1995 to 2013, we selected cases in 13 SEER areas to make the population more consistent over time between the two datasets.

### Study design and study population

This is a retrospective cohort study. We identified all newly diagnosed adult MCL patients residing in Texas and in 13 U.S. SEER areas from 1995 to 2013 recorded in TCR and SEER databases. Patients were included if they: (a) had a date of initial MCL diagnosis in 1995 or later; (b) had a primary diagnosis of MCL (International Classification of Diseases for Oncology 3^rd^ edition [ICD-O-3] site code: 9673); and (c) were 20 or older at the time of diagnosis.

### Study variables

### Main exposure variables

The main exposure variables are year of diagnosis defined as four calendar year periods (1995–1999, 2000–2003, 2004–2008, 2009–2013) and geographic areas (Texas and 13 SEER areas). The incidence rates of MCL cancer were compared among those four time periods and by geographic areas.

### Main outcome variables

The primary outcome variable was cumulative MCL incidence rate in Texas and SEER areas from 1995 to 2013. IRs were expressed as number of new cases per 100,000 persons per year in a certain time period and were standardized to the 2000 U.S. population.

### Other variables

Covariates included age at diagnosis, gender, race/ethnicity, and tumor stage. Age was classified according to five categories with < 50, 50–59, 60–69, 70–79 and ≥ 80 years. Gender was a binary variable with male and female. Race/ethnicity was categorized as non-Hispanic white, non-Hispanic black, Hispanics, and other. Lymphoma stage was classified as localized, regional, advanced (Ann Arbor stages 3–4, distant stage recorded in SEER) and unknown stages.

### Statistical analysis

Relative risk was expressed as the ratio of incidence rates between two comparison groups. Incidence rates were stratified by age group, gender, race/ethnicity, tumor stage and region (Texas vs. SEER areas) and were plotted against diagnostic periods. Annual percent changes in incidence rates were also calculated.

We used negative binomial regression model for count data to assess the association between the year of diagnosis and MCL incidence rates. The negative binomial regression model is an alternative to Poisson regression model when over-dispersion existed in the data. The count data was stratified by diagnostic period, age group, gender, race/ethnicity and tumor stages. Population at risk for each group during each time period was calculated using SEER^*^stat 8.3.4 and was used as an offset in the negative binomial regression after a log transformation [27]. Negative binomial regression was conducted using SAS enterprise guide 7.1. All regression models included age, gender, race/ethnicity and tumor stage as covariates. Subgroup incidence and trend analyses were conducted for high risk groups.

Overall survival for MCL patients in SEER and TCR areas was analyzed and plotted. Cumulative incidence function was also used to calculate the cumulative incidence of death for MCL patients. Cumulative incidence of death for MCL patients was plotted by calendar period of diagnosis and tumor stage.

## CONCLUSIONS

From 1995 to 2013, MCL incidence rates were significantly higher in male, non-Hispanic white, elderly patients with advanced stage tumors in both Texas and SEER areas. MCL incidence rates also increased over time in both Texas and SEER areas, with increases being greater in male, white, elderly patients (aged ≥ 70 years) with advanced stage tumors. When comparing Texas to SEER areas, we found that the increase of MCL incidence rates has slowed down for the past few years, especially in male and patients with advanced stage tumors. In general, Texas has similar MCL incidence trends and disparities as SEER areas, but the incidence rates in Texas were slightly lower than that in SEER areas. Future etiology studies, cancer prevention, and treatment development programs should focus on those high risk populations in both Texas and SEER areas.

## Abbreviations

NHL: non-Hodgkin lymphoma
MCL: mantle cell lymphoma
SEER: Surveillance, Epidemiology and End Results
TCR: Texas Cancer Registry
IR: Incidence rates
RR: Relative risk
APC: annual percent change
ICD-O-3: International Classification of Diseases for Oncology 3rd edition
R-CHOP: rituximab plus cyclophosphamide, doxorubicin, vincristine, and prednisone regimen
